# Supplementary figures and images for: Cooperation with autonomous machines through culture and emotion
Source: PLoS One. 2019 Nov 11;14(11):e0224758. doi: 10.1371/journal.pone.0224758 (PMC6844555; doi:10.1371/journal.pone.0224758)

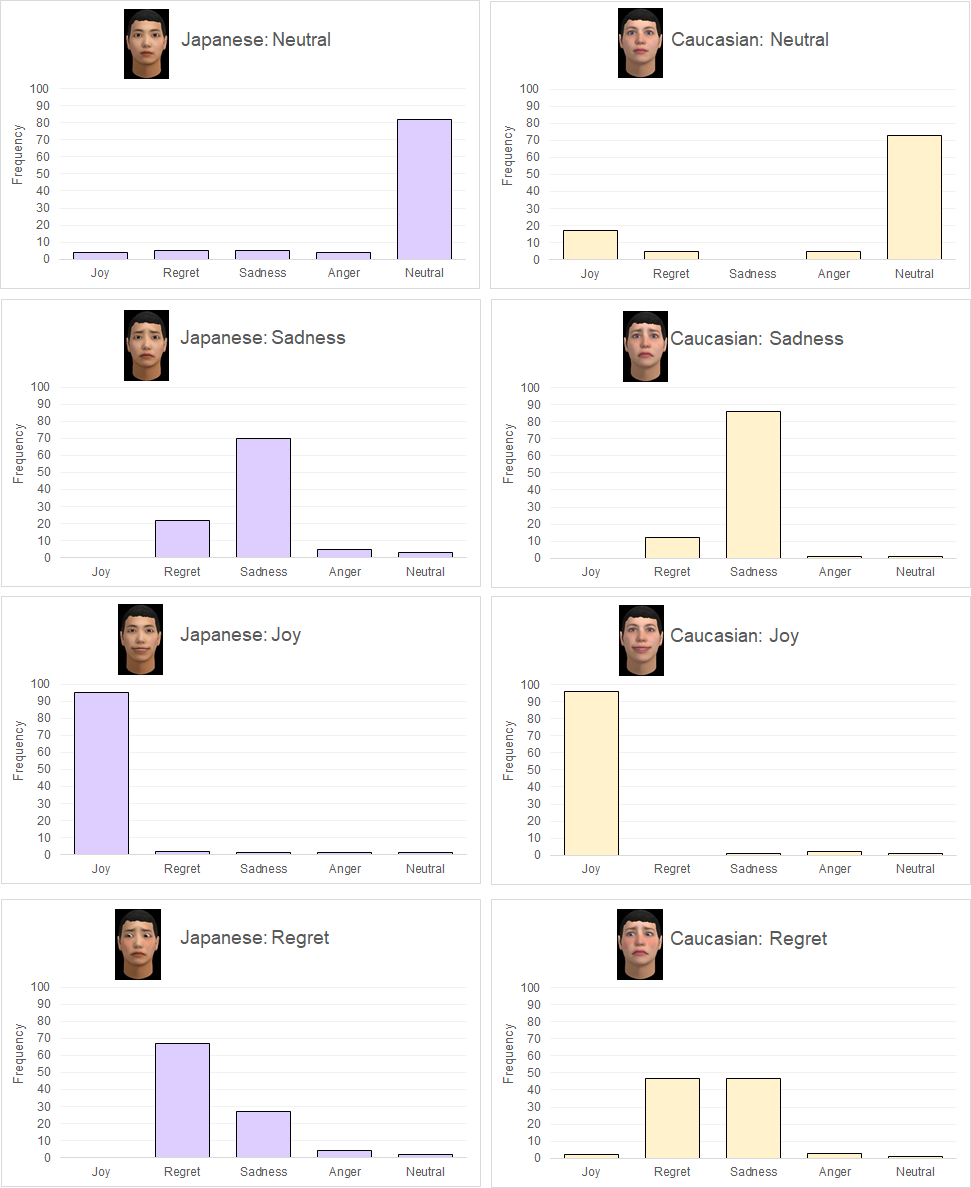

Supplement: S1 Fig — (TIF) [file pone.0224758.s001.tif]

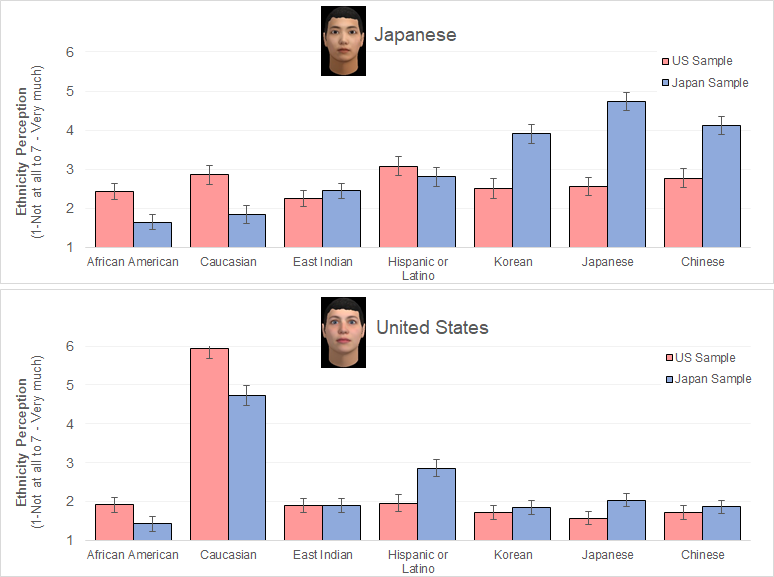

Supplement: S2 Fig — (TIF) [file pone.0224758.s002.tif]

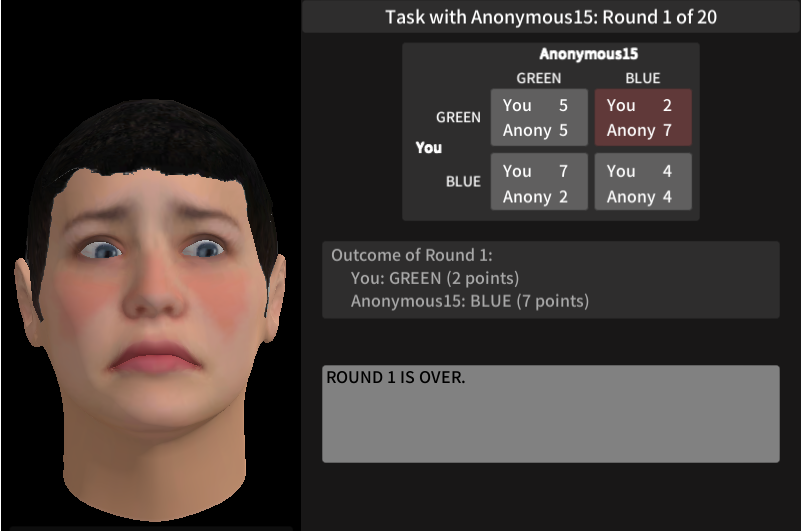

Supplement: S3 Fig — The counterpart in this case has the same culture and is showing cooperative emotions. (TIF) [file pone.0224758.s003.tif]

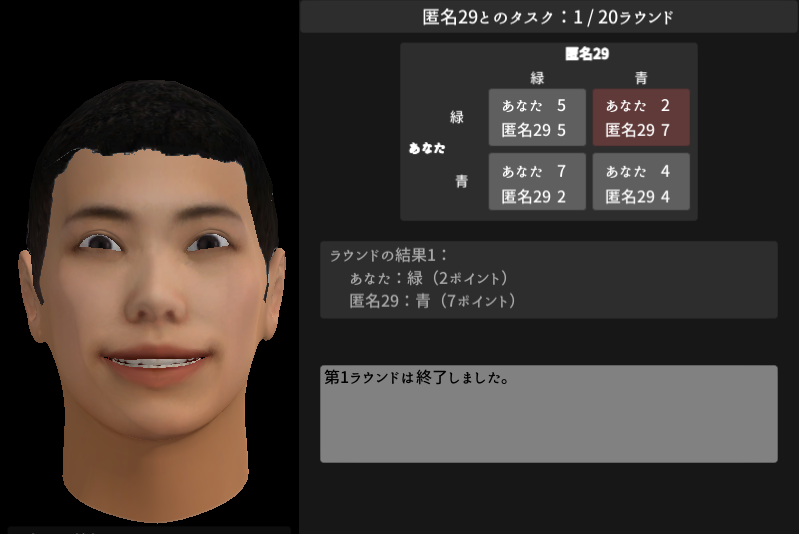

Supplement: S4 Fig — The counterpart in this case has the same culture and is showing competitive emotions. (TIF) [file pone.0224758.s004.tif]
