# Supplementary material for: Cooperation with autonomous machines through culture and emotion
Source: PLoS One. 2019 Nov 11;14(11):e0224758. doi: 10.1371/journal.pone.0224758 (PMC6844555; doi:10.1371/journal.pone.0224758)
Supplement: S1 File — (DOCX) [file pone.0224758.s005.docx]

**Validation experiment for emotion and ethnicity perception in virtual faces**

We conducted an experiment to validate that the virtual faces’ emotion expressions and ethnicities were being perceived as expected. To accomplish this, we recruited a separate sample of participants using online pools: 49 participants from the United States were recruited through Amazon Mechanical Turk; and, 51 participants from Japan were recruited through Yahoo! Japan Crowdsourcing. The demographics for these samples were similar to those for the main experiment.

To validate the emotion expressions, participants were shown pictures of the expressions for neutral, sadness, joy, and regret, in both the Japanese and Caucasian faces. For each picture, they were asked to choose which emotion was best represented from five options: joy, regret, sadness, anger, and neutral. S1 Fig shows the results for every picture. All expressions were most likely to be perceived as the intended emotion: neutral – Japanese face, *χ^2^*(4) = 240.30, *P* < 0.001, Caucasian face, *χ^2^*(3) = 126.72, *P* < 0.001; sadness – Japanese, *χ^2^*(3) = 116.72, *P* < .001, Caucasian, *χ^2^*(4) = 201.68, *P* < .001; joy – Japanese, *χ^2^*(4) = 351.60, *P* < 0.001; Caucasian, *χ^2^*(3) = 268.88, *P* < 0.001; and, regret – Japanese, *χ^2^*(3) = 108.52, *P* < 0.001, Caucasian, *χ^2^*(4) = 121.60, *P* < 0.001.

To validate perception of the face’s ethnicity, participants were shown the picture for the Japanese and Caucasian faces and were asked to rate on a 7-point Likert scale (1, *Not at all*, to 7, *Very much*), how much each face represented the following ethnicities: African American, Caucasian, East Indian, Hispanic or Latino, Korean, Japanese, and Chinese. S2 Fig shows the results for the Japanese and US participant samples. To analyze these data we ran a participant sample (United States vs. Japan; between-participants) × ethnicity perception (African American vs. Caucasian vs. East Indian vs. Hispanic or Latino vs. Korean vs. Japanese vs. Chinese; repeated-measures) mixed ANOVA for the Japanese and the Caucasian faces. For the Japanese face, there was the expected main effect of ethnicity perception, *F*(6, 588) = 16.72, *P* < 0.001, partial η^2^ = 0.146: participants perceived the Japanese face to be most likely from the Japanese ethnicity (though perceptions of a Chinese ethnicity were high as well). Interestingly, there was a participant sample × ethnicity perception interaction, *F*(6, 588) = 16.15, *P* < 0.001, partial η^2^ = 0.141: with participants from the US being less likely to perceive any particular ethnicity in the face than participants from Japan. For the Caucasian face, there was also the expected main effect of ethnicity perception, *F*(6, 588) = 115.51, *P* < 0.001, partial η^2^ = 0.541: participants perceived the Caucasian face to be most likely from the Caucasian ethnicity. There was also a participant sample × ethnicity perception interaction, *F*(6, 588) = 7.66, *P* < 0.001, partial η^2^ = 0.073: with participants from the US rating higher on perceptions of Caucasian ethnicity than participants from Japan.
