# Supplementary material for: Cooperation with autonomous machines through culture and emotion
Source: PLoS One. 2019 Nov 11;14(11):e0224758. doi: 10.1371/journal.pone.0224758 (PMC6844555; doi:10.1371/journal.pone.0224758)
Supplement: S1 Table — (DOCX) [file pone.0224758.s007.docx]

**Table 1. Descriptive statistics for main experiment.**

| Participant Sample | Counterpart Culture | Counterpart Type | Emotion | Mean | Std. Error |
| --- | --- | --- | --- | --- | --- |
| Japan | United  States | Machine | Competitive | .295 | .054 |
|  |  |  | Cooperative | .528 | .057 |
|  |  |  | Neutral | .263 | .058 |
|  |  | Human | Competitive | .326 | .049 |
|  |  |  | Cooperative | .456 | .066 |
|  |  |  | Neutral | .512 | .063 |
|  | Japan | Machine | Competitive | .260 | .062 |
|  |  |  | Cooperative | .434 | .055 |
|  |  |  | Neutral | .428 | .052 |
|  |  | Human | Competitive | .305 | .049 |
|  |  |  | Cooperative | .485 | .051 |
|  |  |  | Neutral | .350 | .050 |
| United States | United  States | Machine | Competitive | .299 | .054 |
|  |  |  | Cooperative | .462 | .051 |
|  |  |  | Neutral | .353 | .062 |
|  |  | Human | Competitive | .296 | .055 |
|  |  |  | Cooperative | .441 | .051 |
|  |  |  | Neutral | .365 | .058 |
|  | Japan | Machine | Competitive | .182 | .056 |
|  |  |  | Cooperative | .437 | .059 |
|  |  |  | Neutral | .362 | .049 |
|  |  | Human | Competitive | .371 | .058 |
|  |  |  | Cooperative | .396 | .057 |
|  |  |  | Neutral | .409 | .058 |
